# BATFNet: Boundary-Aware Transformer Fusion Network for RGB-DSM Semantic Segmentation of Remote Sensing Images

**DOI:** 10.3390/s26103205

**Published:** 2026-05-19

**Authors:** Yilin Tong, Meng Tang, Yu Zhang, Yan Huang, Jing Huang, Yuelin He, Yuxin Liu, Edore Akpokodje, Dan Zheng

**Affiliations:** 1 School of Electronic Engineering, Wuhan Vocational College of Software and Engineering, Wuhan 430205, China; 20260004@whvcse.edu.cn (Y.T.); 42200251@whvcse.edu.cn (Y.H.); 20230028@whvcse.edu.cn (J.H.); 2 Department of Computer Science, Aberystwyth University, Penglais, Aberystwyth SY23 3DB, UK; met57@aber.ac.uk (M.T.); yuh22@aber.ac.uk (Y.H.); yuxinliu25@stu.pku.edu.cn (Y.L.); eta@aber.ac.uk (E.A.); 3 Hubei Institute of Measurement and Testing Technology, Wuhan 430223, China; zhangyu@himtt.net; 4 Department of Computer Science, Peking University, Beijing 100871, China

**Keywords:** semantic segmentation, remote sensing, cross-modal attention, deep learning

## Abstract

Semantic segmentation of very-high-resolution remote sensing imagery benefits from combining RGB appearance with Digital Surface Model (DSM) height information, especially in urban scenes where spectrally similar objects often differ in elevation. On the ISPRS Vaihingen and Potsdam benchmarks, BATFNet achieves mIoU scores of 84.06% and 85.31%, respectively, outperforming representative RGB–DSM fusion baselines on most land-cover categories. BATFNet is a supervised boundary-aware Transformer fusion network that uses DSM-derived edge priors to guide bidirectional cross-modal attention and decoder refinement. With a dual-branch ResNet-50 backbone for modality-specific feature extraction, the proposed framework effectively integrates RGB and DSM information while recovering fine spatial details. These results show that exploiting DSM-derived structural cues improves boundary delineation and reduces confusion among spectrally similar urban classes.

## 1. Introduction

Semantic segmentation of very high-resolution remote sensing imagery aims to assign a semantic label to each pixel and is an important task in urban land-cover analysis [1,2]. However, urban aerial scenes remain challenging because buildings, roads, vegetation, and vehicles often exhibit strong inter-class spectral similarity and substantial intra-class variation, making reliable interpretation from RGB imagery alone difficult [3,4,5].

However, in contrast to natural scene imagery, remote sensing images pose significantly more complex challenges for semantic segmentation. Different categories of ground objects often exhibit a high degree of spectral similarity in optical imagery, with buildings and roads frequently sharing highly similar color and textural characteristics, and impervious surfaces often being difficult to distinguish from parking lots on the basis of spectral information alone. Meanwhile, objects within the same category may display marked variability due to differences in material properties, orientation, illumination conditions, and seasonal effects [3,6]. Furthermore, the nadir perspective and varying imaging conditions in aerial photography further exacerbate the difficulty of recognition, rendering conventional approaches based on single-modality optical data inadequate for handling the complexity and diversity of ground objects distributions [4,5].

Integrating RGB imagery with Digital Surface Model (DSM) data has been widely recognized as an effective strategy for improving semantic segmentation in remote sensing.RGB imagery provides rich spectral and textural information, while DSM data contributes complementary elevation and geometric cues. This synergy is particularly valuable in complex urban environments, where spectrally similar land-cover classes can nonetheless exhibit pronounced differences in elevation. For instance, buildings and impervious surfaces often share comparable spectral characteristics yet differ markedly in height. Likewise, the incorporation of height information enables more reliable discrimination between trees and low-lying vegetation, which frequently present overlapping spectral signatures in conventional optical imagery alone [7,8]. Accordingly, this study treats DSM data not as a passive auxiliary input, but as a structurally informative modality that drives cross-modal fusion and contributes to precise boundary refinement.

Recent advances in deep learning have driven remarkable progress in semantic segmentation, particularly for high-resolution remote sensing imagery. Fully Convolutional Networks (FCNs) [9] pioneered end-to-end pixel-wise prediction, marking a paradigm shift from handcrafted features to learned representations. Subsequently, encoder–decoder architectures represented by U-Net [10,11] improved the recovery of spatial details through multi-level skip connections, whereas the DeepLab family of methods [12,13] leveraged atrous convolutions combined with ASPP modules to effectively extract contextual information at multiple scales. PSPNet [14] fused multi-scale context through pyramid pooling modules. A fundamental drawback of CNNs, however, lies in their reliance on local receptive fields, which limits the modeling of long-range dependencies and holistic contextual information [15]. To overcome this limitation, Vision Transformers (ViTs) [16,17] and hierarchical variants such as Swin Transformer [18] and SegFormer [19] have demonstrated strong capability in modeling global dependencies through self-attention mechanisms. More recently, the Mamba architecture based on State Space Models (SSMs) [20] has emerged as a computationally efficient alternative, realizing long-range dependency modeling while maintaining linear complexity [21,22]. Despite these advances, most existing methods remain confined to single-modality inputs, which restricts their ability to exploit the information provided by multiple data sources in remote sensing. In this study, the term multimodal refers to the joint use of heterogeneous data modalities that describe the same scene from different but complementary perspectives. Specifically, RGB imagery conveys spectral and textural information, whereas DSM data provides elevation and structural cues. Accordingly, multimodal fusion has become an important direction in remote sensing semantic segmentation. However, effectively integrating RGB imagery and DSM data remains a challenging and unresolved problem. Current fusion approaches can broadly be classified into three types [23]: (1) Early fusion methods directly concatenate RGB and DSM channels at the input stage [24], thereby treating the two modalities as a unified multi-channel representation. Although straightforward, this strategy implicitly assumes that all input channels can be processed in the same manner and fails to account for the distinctive properties of each modality, which may lead to mutual interference between the two data sources. (2) Late fusion methods employ separate encoders for different modalities and combine predictions or high-level features only at the decision stage. However, because interaction between modalities is absent during feature extraction, such methods are unable to exploit complementary cross-modal cues at intermediate representation levels. (3) Feature-level fusion approaches, exemplified by FuseNet [25] and V-FuseNet [7], attempt to combine features at multiple encoder stages through element-wise addition or channel concatenation. Although attention mechanisms such as SE-Net [26] and CBAM [27] have shown promise in feature refinement, they were primarily designed for within-modality recalibration and do not explicitly address the cross-modal interactions between RGB and DSM representations. Recent works including TMFNet [28], LMFNet [29], and MSFFNet [30] have proposed a variety of multimodal fusion frameworks, some of which incorporate Transformer architectures to enhance feature interaction. However, effectively integrating RGB and DSM features while adequately capturing long-range dependencies between modalities remains insufficiently explored.

To address these limitations, we propose BATFNet, a boundary-aware Transformer fusion network for urban RGB–DSM semantic segmentation. BATFNet uses DSM-derived edge priors to guide bidirectional cross-modal interaction and decoder refinement, thereby enhancing the recovery of fine structures while preserving modality-specific representations. The primary contributions of this work can be highlighted as follows:

- We propose a boundary-aware cross-modal fusion module that incorporates DSM-derived edge priors into bidirectional RGB–DSM attention, enabling more effective multimodal interaction around elevation discontinuities.
- We present a cross-modal fusion network that processes RGB and DSM data through dedicated encoding pathways, allowing the model to fully exploit modality-specific representations while achieving effective inter-modal feature integration.
- Experiments on the ISPRS Vaihingen and Potsdam benchmarks demonstrate that BATFNet consistently outperforms representative RGB–DSM baselines, particularly for classes with complex visual characteristics.

## 2. Related Work

### 2.1. CNN-Driven Semantic Segmentation in Remote Sensing Imagery

Semantic segmentation in remote sensing has undergone a paradigm shift with the advent of deep learning, transitioning from hand-crafted feature representations to end-to-end trainable architectures. As a pioneering framework for semantic segmentation, FCN [9] enabled end-to-end pixel-wise prediction by replacing fully connected layers with convolutional layers, thereby establishing a new paradigm for CNN-based image segmentation. This foundational architecture was subsequently refined by numerous variants tailored for remote sensing applications. U-Net [10], which was initially proposed for the segmentation of biomedical images, established symmetric skip pathways linking the encoder and decoder stages to retain fine-grained spatial details. Owing to its strong capability in integrating low-level spatial cues with high-level semantic representations, U-Net has been extensively applied to remote sensing segmentation tasks [2,31]. SegNet [32] introduced an efficient encoder-decoder architecture that employs pooling indices for upsampling, thus decreasing the model’s parameter count without sacrificing segmentation performance.

Beyond encoder–decoder designs, subsequent research has also focused on mitigating the spatial information loss inherent in hierarchical downsampling, and improving segmentation quality by expanding receptive fields and leveraging multi-scale feature representations [33]. The DeepLab family [12,13] adopted dilated convolutions to expand the receptive field and subsequently proposed Atrous Spatial Pyramid Pooling (ASPP) for aggregating contextual information at multiple scales while preserving spatial resolution. PSPNet [14] utilized pyramid pooling modules to integrate multi-scale contextual representations, enabling more comprehensive feature extraction across varying scales. Within the remote sensing domain, ABCNet [34] incorporated bilateral contextual attention mechanisms to strengthen global semantic representation, whereas SFFNet [35] exploited wavelet-based features in both the spatial and frequency domains to refine boundary delineation. MAResU-Net [2] enhanced the joint modeling of local details and global semantics via multi-stage attention modules, delivering competitive performance across multiple benchmark datasets.

While these methods, along with approaches such as ABCNet and SFFNet, have attempted to broaden the effective receptive field through various strategies, their ability to capture long-range dependencies remains fundamentally bounded by the local nature of convolution operations [5,15]. This limitation has prompted researchers to seek alternative architectures capable of more effective global dependency modeling.

### 2.2. Transformer and Mamba Architectures for Remote Sensing Image Semantic Segmentation

The remarkable achievements of Transformers in natural language processing [36] have catalyzed their broad adoption in the computer vision community [37,38]. Vision Transformer (ViT) [16] pioneered the application of Transformer architectures to visual recognition, leveraging self-attention to inherently support global dependency modeling and enabling effective capture of long-range spatial relationships in segmentation tasks. Building upon this foundation, the Swin Transformer [18] struck an effective trade-off between computational efficiency and representation power by introducing hierarchical feature maps and shifted window-based self-attention, and has since become a dominant backbone for dense prediction. Within the remote sensing field, Wang et al. [39] proposed UNetFormer, which employs the Swin Transformer as an encoder backbone and integrates global-local attention mechanisms to deliver balanced accuracy across diverse land-cover categories. CMTFNet [38] enhanced inter channel feature interaction to enrich contextual representations. The hybrid model TransUNet [40] unifies the fine-grained local feature extraction capability of CNNs with the global reasoning power of Transformers, demonstrating strong results in remote sensing segmentation. LSRFormer [41] augments each stage of a convolutional backbone with efficient long-short range Transformer modules to complement global semantic information. Despite the evident strengths of Transformer-based approaches in global context modeling and multi-scale feature integration, their substantial computational overhead and demanding training requirements continue to hinder deployment in large-scale applications [42].

More recently, the Mamba architecture has emerged as a promising alternative for visual sequence modeling. Built upon the State Space Model (SSM) framework, Mamba achieves effective long-range dependency capture while preserving linear computational complexity [20]. In contrast to the self-attention mechanism employed by Transformers, Mamba offers reduced memory consumption and accelerated inference for lengthy sequences, which makes it especially appealing for segmenting large-scale high-resolution imagery [21,22]. For semantic segmentation, Mamba models’ architectures can efficiently encode spatial representations through state transitions and input projections, and their integration with convolutional operations further strengthens the perception of local contextual patterns [43,44]. For instance, RS3Mamba [21] realizes global representation learning and efficient remote sensing classification by incorporating SSM with a dynamic multi-path activation strategy. CM-UNet [22] integrates CNN and Mamba modules into a unified hybrid framework for remote sensing semantic segmentation. Despite the significant progress achieved by Transformer and Mamba-based architectures, these methods predominantly operate on single-modality RGB inputs, leaving the complementary potential of multimodal remote sensing data largely unexploited. Extending these architectures to effectively leverage heterogeneous data sources remains an open and critical research challenge.

### 2.3. Multi-Modal Fusion for Remote Sensing

To overcome the representational bottleneck of single-modality inputs, multi-modal data fusion has emerged as a key research direction in remote sensing, aiming to jointly exploit the complementary strengths of diverse data sources [23]. In remote sensing, the fusion of optical imagery and elevation data (DSM) has attracted particular attention, as these two modalities provide fundamentally complementary information: optical imagery captures spectral appearance while DSM encodes geometric structure. Depending on the stage at which information from different modalities is integrated, fusion strategies can generally be classified into three categories: early fusion at the input level, middle fusion at the feature level, and late fusion at the decision level.

Input-level fusion represents the most straightforward approach, where data from multiple modalities are concatenated along the channel dimension before being fed into a single-stream network. For instance, Sun and Wang [24] directly combined RGB and DSM channels to construct a multi-channel input for FCN-based segmentation of very-high-resolution remote sensing images. Despite its simplicity, this approach processes all input channels uniformly, making it difficult to capture the distinct statistical and semantic characteristics of each modality and thereby often producing suboptimal feature representations. To address this limitation, researchers have increasingly turned to feature-level fusion strategies, which allow modality-specific representations to be learned independently before cross-modal interaction is performed during feature extraction. FuseNet [25] proposed a pioneering dual-branch encoder architecture where features from the depth branch are progressively fused into the RGB branch through element-wise addition at each encoder stage. Audebert et al. [7] extended this concept with V-FuseNet, which investigated various fusion architectures for combining RGB and additional spectral or elevation data in urban remote sensing, demonstrating significant improvements over single-modality baselines on the ISPRS benchmarks. Sherrah [8] demonstrated that incorporating normalized DSM (nDSM) alongside RGB channels substantially improves segmentation performance for high-resolution aerial imagery. Paisitkriangkrai et al. [45] incorporated deep CNN features, manually designed features, and elevation information into a conditional random field framework for pixel-wise labeling. At the other end of the fusion spectrum, decision-level methods combine the outputs of independently trained modality-specific networks through strategies such as voting, averaging, or learnable weighting. This late fusion paradigm, however, precludes cross-modal interaction during feature learning, limiting its capacity to exploit the complementary information that resides at intermediate feature levels.

With the emergence of Transformer-based architectures, cross-attention has provided a natural mechanism for multimodal fusion, where queries originate from one modality and keys and values from another, enabling explicit interactions between heterogeneous representations. Representative works include SA-Gate [46], which disentangles and aggregates modality-specific features through a learned gating mechanism for RGB-D segmentation; CMX [47], which facilitates bidirectional information exchange between RGB and auxiliary modality branches within a Transformer framework; and TokenFusion [48], which dynamically replaces uninformative tokens with projected tokens from complementary modalities. More recently, several studies have explored RGB-DSM fusion tailored to remote sensing scenarios. TMFNet [28] introduced a Transformer-based fusion module combined with border-region attention for cross-level feature integration. LMFNet [29] proposed a lightweight shared-weight Vision Transformer for joint processing of RGB, NirRG, and DSM data. MSFFNet [30] explored frequency-domain fusion via wavelet decomposition to preserve edge information during RGB-DSM integration. Despite these efforts, most existing methods either originate from indoor RGB-D settings or adopt relatively simple fusion schemes that do not adequately account for the unique properties of aerial DSM data, where elevation is continuous rather than discrete and spatial patterns differ fundamentally from those in ground-level imagery. These observations motivate the design of a dedicated cross-modal fusion mechanism specifically tailored to RGB-DSM fusion in aerial remote sensing.

## 3. Datasets and Evaluation Metrics

### 3.1. ISPRS Vaihingen Dataset

The ISPRS Vaihingen dataset [49] serves as one of the most established benchmarks for semantic segmentation in urban aerial scenes. It has been extensively adopted as a benchmark dataset in remote sensing semantic segmentation and multimodal urban scene understanding studies, enabling direct comparison across methods under widely shared evaluation protocols [3,7,8]. It was collected over the city of Vaihingen in southern Germany, a relatively small urban area characterized by a mixture of residential buildings, streets, and vegetation. The dataset comprises 33 image tiles of varying sizes, with an average size of approximately 2494×2064 pixels. Each tile comprises three spectral channels, namely near-infrared (NIR), red (R), and green (G), and is provided as a true orthophoto (TOP) with a ground sampling distance (GSD) of 9 cm. The dataset is further accompanied by a co-registered DSM produced via dense image matching, supplying elevation information at the same ground spatial resolution. Pixel-level annotations are provided for six land-cover categories, namely impervious surfaces, buildings, low vegetation, trees, cars, and clutter/background. The class distribution is imbalanced, with impervious surfaces and buildings accounting for the largest proportions, whereas cars and clutter/background constitute minority classes. Following the standard evaluation protocol adopted by the majority of previous studies [7,8], we employed the same training strategy. For model training, both the optical images and the associated DSM tiles are divided into 512×512 pixel patches, and data augmentation strategies such as random flipping, scaling, and rotation are applied to enrich the diversity of training samples.

### 3.2. ISPRS Potsdam Dataset

The ISPRS Potsdam dataset [49] is another prominent benchmark provided by the ISPRS Commission, acquired over the city of Potsdam, the capital of Brandenburg, Germany. Along with the Vaihingen dataset, the ISPRS Potsdam dataset has been widely adopted as a benchmark for evaluating high-resolution urban semantic segmentation methods, especially those based on RGB and DSM fusion [3,7,8]. In comparison with Vaihingen, the Potsdam scene encompasses a broader and more heterogeneous urban landscape, featuring a greater proportion of historic architecture, spacious streets, and dense vegetated areas, thereby offering a richer pool of training samples that more thoroughly captures the variability across different land-cover types. This dataset consists of 38 image tiles, each of a fixed dimension of 6000×6000 pixels. Each tile comprises four spectral channels—red (R), green (G), blue (B), and near-infrared (NIR)—captured at a ground sampling distance (GSD) of 5 cm, yielding a finer spatial resolution compared to the Vaihingen dataset. Furthermore, a co-registered digital surface model (DSM) is supplied for every tile at the matching spatial resolution. The labeling protocol follows the same convention as the Vaihingen dataset, encompassing the identical six land-cover categories. In accordance with the standard experimental setup adopted in prior studies [3,7], we apply the same training configuration. The input images and DSM data are partitioned into 512×512 patches for training, in line with the Vaihingen setting. The Vaihingen and Potsdam benchmarks offer complementary urban evaluation settings in terms of spatial resolution, scene heterogeneity, and land-cover composition. They therefore provide a suitable basis for fair comparison under a shared benchmark protocol.

A summary of the principal properties of the two datasets is presented in Table 1.

### 3.3. Evaluation Metrics

To quantitatively assess segmentation performance, three widely used metrics were adopted, namely Overall Accuracy (OA), the F1-score for each class, and mean Intersection over Union (mIoU).

Overall Accuracy (OA) measures the proportion of correctly classified pixels over the entire test set:

(1) $$\mathrm{OA}=\frac{\sum_{i=1}^{C}{\mathrm{TP}}_{i}}{\sum_{i=1}^{C}\left({\mathrm{TP}}_{i}+{\mathrm{FP}}_{i}+{\mathrm{FN}}_{i}+{\mathrm{TN}}_{i}\right)},$$

where *C* denotes the number of classes, and ${\mathrm{TP}}_{i}$, ${\mathrm{FP}}_{i}$, ${\mathrm{FN}}_{i}$, and ${\mathrm{TN}}_{i}$ represent the numbers of true positives, false positives, false negatives, and true negatives for class *i*, respectively. Although OA provides an intuitive measure of overall segmentation performance, it may be biased toward classes occupying relatively large image areas.

To provide a more detailed class-wise evaluation, the F1-score was reported for each category. The F1-score for class *i* is defined as the harmonic mean of precision and recall:

(2) $$F1_{i}=\frac{2\;{\mathrm{TP}}_{i}}{2\;{\mathrm{TP}}_{i}+{\mathrm{FP}}_{i}+{\mathrm{FN}}_{i}}.$$

This metric jointly accounts for false positives and false negatives and therefore provides a balanced assessment of segmentation performance for individual classes. In addition, mean Intersection over Union (mIoU) was used to evaluate the overlap between the predicted segmentation results and the ground-truth labels:

(3) $$\mathrm{mIoU}=\frac{1}{C^{\prime }}\sum_{i=1}^{C^{\prime }}\frac{{\mathrm{TP}}_{i}}{{\mathrm{TP}}_{i}+{\mathrm{FP}}_{i}+{\mathrm{FN}}_{i}},$$

where $C^{\prime }$ denotes the number of evaluated classes. As a widely used metric in semantic segmentation, mIoU provides a rigorous assessment of segmentation quality by accounting for both false expansion and false omission in the predicted regions.

Collectively, OA, class-wise F1-scores, and mIoU provide a comprehensive evaluation of the proposed method at both the overall and category-specific levels.

## 4. Proposed Method

### 4.1. Overall Architecture

**Figure 1:**
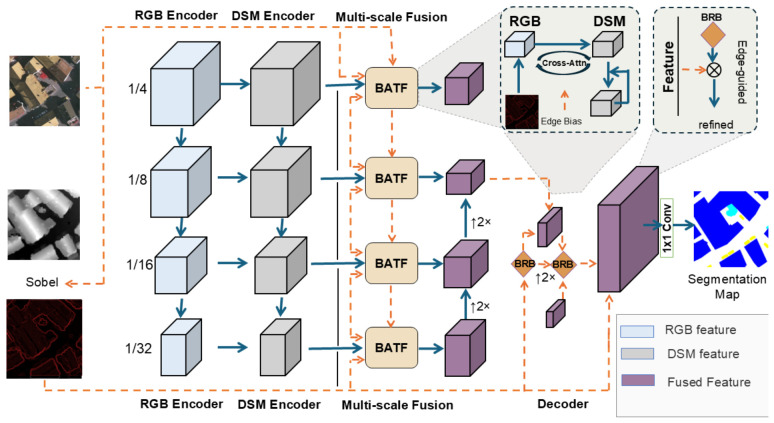
Overall architecture of BATFNet. RGB and DSM features are extracted by two modality-specific encoders, fused at multiple scales by boundary-aware Transformer fusion (BATF) blocks, and decoded through boundary refinement blocks to generate the final segmentation map.

As illustrated in Figure 1, BATFNet receives an RGB image. $I_{\mathrm{rgb}}\in R^{Copt\times H\times W}$ and its corresponding DSM $I_{\mathrm{dsm}}\in R^{1\times H\times W}$, and produces a pixel-wise classification map Y^∈RC×H×W, where *C* denotes the number of land cover categories. The architecture comprises three components: (1) a dual-stream encoder designed to extract features from each modality at multiple scales; (2) a cross-modal fusion module with boundary guidance that enables bidirectional interaction between RGB and DSM features at deeper semantic stages; and (3) a decoder built upon a feature pyramid network and equipped with progressive boundary refinement to recover fine spatial details. The DSM provides explicit geometric information about scene structure, and abrupt changes in elevation often correspond to object boundaries that may not be clearly distinguishable in RGB imagery. To exploit this property, we extract an edge prior map $E_{d}\in R^{1\times H\times W}$ from the DSM and use it to guide both cross-modal fusion and boundary refinement in the decoder. Specifically, the horizontal and vertical gradients, denoted by $G_{x}$ and $G_{y}$, are computed by convolving $I_{\mathrm{dsm}}$ with the Sobel operators. The edge prior map is then obtained from the gradient magnitude and normalized to the range [0,1]:

(4) $$E_{d}=\mathrm{MinMaxNorm}\;\left(\sqrt{G_{x}^{2}+G_{y}^{2}}\right),$$

Two independent ResNet-50 [50] encoders are employed to extract hierarchical features from the RGB image and the DSM, respectively. The RGB encoder directly processes the three-channel optical image. In the DSM branch, the single-channel elevation map is concatenated with the edge prior $E_{d}$ and then projected into a three-channel representation through a 1×1 convolutional layer followed by batch normalization and a nonlinear activation function. This design allows the encoder to exploit both the original elevation information and the boundary cues derived from the DSM from the initial stage of feature extraction:

(5) $$X_{\mathrm{dsm}}={\mathrm{ConvBNReLU}}_{1\times 1}\;\left(\mathrm{Cat}(I_{\mathrm{dsm}},\;E_{d})\right).$$

At the core of BATFNet is a cross-modal fusion module guided by DSM boundary cues, which is introduced at Stage 3 and Stage 4 of the dual-stream encoder. Its purpose is to promote information exchange between RGB and DSM features while emphasizing regions with clear elevation discontinuities. Given a query feature map $F_{q}$ from one branch and a source feature map $F_{s}$ from the other, the query, key, and value representations are defined as

(6) $$Q=W_{q}\;\mathrm{GN}\left(F_{q}\right),\;K=W_{k}\;\mathrm{GN}\left(F_{s}\right),\;V=W_{v}\;\mathrm{GN}\left(F_{s}\right),$$

where $W_{q}$, $W_{k}$, and $W_{v}$ are learnable projections. The resulting features are then divided into multiple attention heads for cross-modal attention computation.

To incorporate structural cues from the DSM, the edge prior $E_{d}$ is first resized to match the spatial resolutions of the query and key features:

(7) $$E_{q}={\mathrm{Up}}_{q}\left(E_{d}\right),\;E_{k}={\mathrm{Up}}_{k}\left(E_{d}\right),$$

where ${\mathrm{Up}}_{q}\left(\cdot \right)$ and ${\mathrm{Up}}_{k}\left(\cdot \right)$ denote bilinear interpolation to the query and key resolutions, respectively. The resized edge priors are then projected into head-wise attention bias maps through two independent learnable 3×3 convolutions:

(8) $$B_{q}=\phi_{q}\left(E_{q}\right),\;B_{k}=\phi_{k}\left(E_{k}\right),$$

where $\phi_{q}\left(\cdot \right)$ and $\phi_{k}\left(\cdot \right)$ map the single-channel edge prior to the bias space of the attention heads. The cross-modal attention is then computed as:

(9) $$\mathrm{Attn}=\mathrm{Softmax}\;\left(\frac{QK^{⊤}}{\sqrt{d_{h}}}+\alpha \cdot \left(B_{q}+B_{k}\right)\right)V,$$

where α is a learnable scalar parameter that controls the overall contribution of the edge-guided bias. In this way, $E_{d}$ serves as an explicit fixed edge prior, while its mapping into the attention bias space is learnable, allowing different attention heads to adapt to different edge-sensitive patterns. This design guides the attention mechanism toward regions with pronounced elevation changes, thereby improving the discrimination of categories with similar spectral characteristics.

Cross-modal attention is embedded in residual transformer blocks and performed bidirectionally at each fusion stage to promote mutual interaction between RGB and DSM features. To preserve the geometric cues carried by the DSM branch, a learnable coefficient is used to regulate its feature update. Let ${\overset{˜}{F}}_{\mathrm{rgb}}$ and ${\overset{˜}{F}}_{\mathrm{dsm}}$ denote the enhanced RGB and DSM features after bidirectional interaction. These two feature maps are then concatenated and refined by two 3×3 convolutional layers to produce the fused representation:

(10) $$F_{\mathrm{fused}}={\mathrm{Conv}}_{3\times 3}\;\left({\mathrm{Conv}}_{3\times 3}\;\left(\mathrm{Cat}({\overset{˜}{F}}_{\mathrm{rgb}},\;{\overset{˜}{F}}_{\mathrm{dsm}})\right)\right).$$

Following cross-modal fusion, the fused high-level features together with the shallow RGB features are fed into an FPN decoder to progressively recover spatial details. The decoder aggregates multi-scale features in a top-down manner:

(11) $$P_{4}=L_{4}\left(F_{4}\right),\;P_{i}={\mathrm{Conv}}_{3\times 3}\;\left(L_{i}\left(F_{i}\right)+\mathrm{Up}\left(P_{i+1}\right)\right),\;i=3,2,1,$$

The resulting pyramid features are then merged to obtain the decoder representation. To improve boundary delineation, the merged feature is progressively refined under the guidance of the DSM edge prior:

(12) $$F_{\mathrm{gated}}=F+F⊙G,\;F_{\mathrm{out}}=\mathrm{ReLU}\;\left(F_{\mathrm{gated}}+{\mathrm{Conv}}_{3\times 3}\;\left({\mathrm{ConvBNReLU}}_{3\times 3}\left(F_{\mathrm{gated}}\right)\right)\right),$$

where G is a spatial gate derived from the DSM edge prior. The refined feature is then fed into a 1×1 classifier to produce the final segmentation map, thereby preserving boundary cues during decoding.

### 4.2. Loss Function

To jointly improve pixel-level classification accuracy and region-level segmentation quality, a composite loss function combining the cross-entropy loss and the Dice loss was adopted:

(13) $$L_{\mathrm{total}}=\lambda_{\mathrm{ce}}\;L_{\mathrm{ce}}+\lambda_{\mathrm{dice}}\;L_{\mathrm{dice}},$$

where $\lambda_{\mathrm{ce}}$ and $\lambda_{\mathrm{dice}}$ denote the weighting coefficients of the cross-entropy and Dice losses, respectively. In this paper, both $\lambda_{\mathrm{ce}}$ and $\lambda_{\mathrm{dice}}$ were set to 0.5, thereby assigning equal weights to the cross-entropy loss and the Dice loss in the overall optimization objective.

For a multi-class segmentation task with *C* classes and *N* pixels, the cross-entropy loss is formulated as

(14) $$L_{\mathrm{ce}}=-\frac{1}{N}\sum_{n=1}^{N}\sum_{c=1}^{C}y_{n,c}\log p_{n,c},$$

where $y_{n,c}$ is the ground-truth indicator for whether pixel *n* belongs to class *c*, and $p_{n,c}$ represents the predicted probability for class *c* at pixel *n*. The cross-entropy term provides direct supervision at the pixel level and encourages the network to learn discriminative category-specific representations. To further enhance the overlap between the predicted regions and the ground-truth annotations, we additionally introduce the Dice loss, which is defined as

(15) $$L_{\mathrm{dice}}=1-\frac{1}{C}\sum_{c=1}^{C}\frac{2\sum_{n=1}^{N}p_{n,c}y_{n,c}+\epsilon }{\sum_{n=1}^{N}p_{n,c}+\sum_{n=1}^{N}y_{n,c}+\epsilon },$$

where ϵ is a small constant introduced to avoid numerical instability. Unlike cross-entropy, which treats each pixel independently, the Dice loss measures the similarity between the predicted mask and the ground-truth region at the class level. This property makes it particularly effective for alleviating the influence of class imbalance and improving the segmentation of minority classes or small objects.

By combining these two objectives, the proposed loss function exploits their complementary advantages. Specifically, the cross-entropy loss promotes reliable pixel-wise classification, whereas the Dice loss emphasizes structural consistency and region overlap. Their combination therefore enables the network to achieve more balanced optimization, leading to improved overall segmentation performance, especially in remote sensing scenes characterized by severe class imbalance and large variations in object scale.

## 5. Results

### 5.1. Implementation Details

All experiments were conducted using PyTorch 2.7.1 on a single Intel Data Center GPU Max 1550 accelerator. To improve training efficiency while reducing memory consumption, Intel Extension for PyTorch (IPEX) was employed to enable automatic mixed-precision training with the bfloat16 format. Both encoder branches use ResNet-50 [50] as the backbone and are initialized with ImageNet pre-trained weights.The model has approximately 67.63 M trainable parameters. The model was trained for 100 epochs using the AdamW optimizer [51], with an initial learning rate of $1\times 10^{-4}$ and a weight decay of $1\times 10^{-4}$. The learning rate was linearly warmed up over the first 5 epochs and subsequently decayed to $1\times 10^{-6}$ following a cosine annealing schedule. Gradient clipping was applied with a maximum norm of 1.0. To alleviate class imbalance, frequency-based class weights were computed from the training set and incorporated into the cross-entropy loss, with a smoothing exponent of 0.5 and an upper bound of 3.0. For data preprocessing, RGB images were first rescaled to 0,1 and then normalized using the channel-wise mean and standard deviation of ImageNet. DSM patches were standardized on a per-patch basis to have zero mean and unit variance. During training, the RGB images and DSM data were jointly augmented by random horizontal flipping, vertical flipping, and random rotation by multiples of $90^{\circ }$. In addition, color jitter and Gaussian noise were applied only to the RGB input to improve robustness to spectral variation. The batch size was set to 8. All experiments used a fixed random seed of 42.

### 5.2. Comparison Methods

Table 2 and Table 3 compare the proposed method with eight baselines on the ISPRS Vaihingen and Potsdam datasets, including the unimodal UNet and seven representative multimodal baselines.

As shown in Table 2, the proposed method achieves an OA of 92.41% and an mIoU of 84.06%, ranking first among all compared methods. Compared with PACSCNet, which attains the second-best mIoU, our method improves mIoU by 0.68 percentage points and OA by 0.76 percentage points. At the class level, the largest improvement is observed for Low Vegetation, whose F1-score is 1.26 percentage points higher than that of PACSCNet. This result indicates that the proposed method is particularly effective in distinguishing categories whose boundaries are difficult to identify from RGB imagery alone but can be better separated with the aid of elevation information from the DSM. Improvements are also observed for Impervious Surface, Building, and Tree, with F1-score gains of 0.28, 0.43, and 0.18 percentage points, respectively. By contrast, PACSCNet performs slightly better on the Car category, achieving an F1-score of 89.80%, whereas our method attains 89.34%. This may be because cars usually occupy only a small proportion of each patch and exhibit limited elevation variation, which reduces the contribution of boundary-guided fusion for this category. Furthermore, all multimodal methods surpass the unimodal UNet by a considerable margin, reinforcing the value of incorporating DSM information for urban scene segmentation.

A similar trend can be observed on the Potsdam dataset, as shown in Table 3. The proposed method achieves the best performance on all evaluation metrics, with an OA of 90.43% and an mIoU of 85.31%. Compared with ACNet and PACSCNet, both of which obtain an mIoU of 84.61%, our method improves mIoU by 0.70 percentage points. In addition, the proposed method attains the highest F1-score in all five categories, further demonstrating its consistent advantage across different land-cover types. These results are consistent with those obtained on the Vaihingen dataset and further confirm the effectiveness and robustness of the proposed method. To further evaluate the contribution of the key components in BATFNet, we conducted ablation experiments on the Vaihingen and Potsdam datasets. Specifically, we compared the full model against two reduced variants by separately removing the boundary prior and bidirectional fusion. The corresponding results are reported in Table 4 and Table 5.

The ablation results on both datasets show that each component contributes positively to the final performance. Removing the boundary prior causes the largest drop, reducing mIoU from 84.06% to 83.05% on Vaihingen and from 85.31% to 84.18% on Potsdam, which confirms the importance of DSM-derived boundary guidance. Removing bidirectional fusion also degrades performance, reducing mIoU to 83.35% and 84.50% on Vaihingen and Potsdam, respectively. These results indicate that both the boundary prior and bidirectional cross-modal interaction are beneficial, while their combination yields the best overall performance.

## 6. Discussion

**Figure 2:**
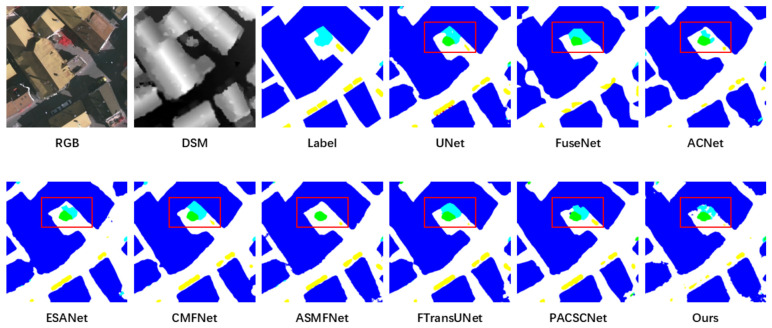
Qualitative comparison of segmentation results on the ISPRS Vaihingen dataset. The first row shows the RGB image, DSM, ground-truth label, and three baseline predictions; the second row shows the remaining baselines and BATFNet. Red boxes highlight regions where boundary delineation differs most clearly.

**Figure 3:**
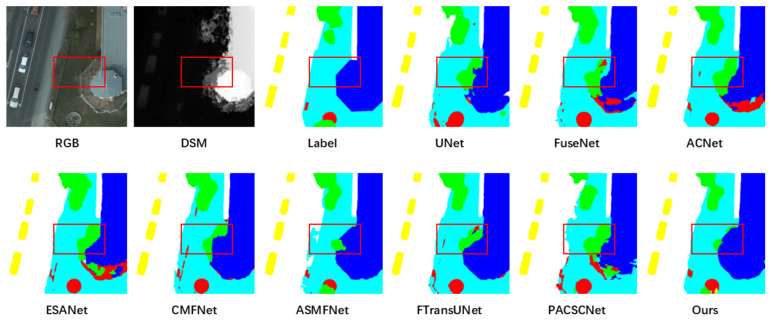
Qualitative comparison of segmentation results on the ISPRS Potsdam dataset. The first row shows the RGB image, DSM, ground-truth label, and three baseline predictions; the second row shows the remaining baselines and BATFNet. Red boxes highlight representative regions with challenging class boundaries.

The qualitative comparisons in Figure 2 and Figure 3 provide further insight into the quantitative results. Compared with the competing methods, BATFNet produces segmentation maps with sharper object boundaries and stronger spatial coherence, particularly in regions where spectral ambiguity coincides with elevation discontinuities. The improvements are especially evident for buildings and low vegetation, for which the proposed method reduces fragmented predictions and yields more regular object contours. This pattern indicates that the proposed boundary-aware fusion strategy is effective in resolving ambiguities among urban classes with similar spectral characteristics.

The observed performance gains are broadly consistent with previous studies reporting that elevation information is beneficial for distinguishing spectrally similar urban classes and for improving boundary localization in high-resolution remote sensing imagery. Earlier RGB-DSM fusion approaches demonstrated the value of incorporating height information for separating impervious surfaces, buildings, and vegetation, while more recent multimodal fusion frameworks further emphasized the importance of effective cross-modal feature interaction. Within this context, the results of the present study suggest that the explicit introduction of DSM-derived boundary priors into both feature fusion and decoder refinement provides an additional mechanism for improving segmentation quality. The contribution of BATFNet therefore lies in extending existing RGB-DSM fusion research through a more targeted use of geometric boundary information during multimodal representation learning.

From an application perspective, the proposed method is particularly relevant to urban remote sensing tasks in which spectral appearance alone is insufficient for reliable class separation. Typical examples include urban land-cover mapping, building and impervious surface extraction, cadastral or land surveying support, and infrastructure monitoring from aerial imagery. In such scenarios, the joint use of RGB imagery and DSM data is valuable because elevation discontinuities often correspond to meaningful object boundaries. At the same time, the effectiveness of the proposed method is influenced not only by network design but also by sensor characteristics and data quality. In practical UAV mapping workflows, accurate co-registration between optical imagery and DSM products is essential, since the proposed fusion mechanism assumes spatial correspondence between spectral patterns and elevation discontinuities. In addition, the reliability of the DSM-derived boundary prior depends on the quality of the elevation data, which may be affected by flight altitude, acquisition geometry, image overlap, dense matching accuracy, and subsequent post processing. These considerations imply that the performance of BATFNet is likely to depend on the quality and consistency of the upstream remote sensing workflow, and that its strongest applicability lies in settings where high-resolution and well-aligned RGB and DSM data are available.

The potential extension of the proposed framework to satellite imagery warrants careful consideration. In principle, the underlying idea of exploiting complementary optical and elevation information remains relevant beyond UAV data. However, compared with high-resolution aerial imagery, satellite data typically exhibit coarser spatial resolution, less distinct object-level boundaries, and less detailed elevation information. These differences may affect the extent to which DSM-guided boundary refinement can provide comparable benefits. Moreover, visually enhanced spatial detail in satellite imagery does not necessarily imply equally reliable geometric structure, which may further limit the effectiveness of the proposed fusion strategy. Accordingly, the applicability of BATFNet to satellite data should be regarded as a promising direction for further study. Several limitations should nevertheless be considered when interpreting the present results. The current evaluation is conducted on the Vaihingen and Potsdam benchmarks. Although these are widely adopted benchmarks in the remote sensing community, they share similar spatial resolutions and scene characteristics, which may not fully reflect the generalization ability of the proposed method to more heterogeneous environments, such as densely built Asian cities or rural areas with irregular structures. Moreover, the relatively modest improvement in the car category indicates that very small objects with limited elevation contrast remain challenging, as their boundary cues in the DSM may be insufficiently distinctive to provide equally strong geometric guidance.

## 7. Conclusions

This paper proposed BATFNet, a boundary-aware Transformer fusion network for RGB-DSM semantic segmentation of high-resolution remote sensing imagery. By incorporating DSM-derived edge priors into bidirectional cross-modal attention and decoder refinement, the proposed method effectively integrates spectral, textural, and elevation information while improving boundary delineation. Experimental results on the ISPRS Vaihingen and Potsdam benchmarks show that BATFNet consistently outperforms representative RGB-DSM fusion baselines in terms of overall segmentation accuracy and individual categories performance. These findings demonstrate the value of boundary-aware multimodal fusion for urban semantic segmentation and confirm the usefulness of explicitly exploiting structural information from DSM data in high-resolution remote sensing image analysis.

Future work will extend the evaluation to more diverse datasets, geographic regions, and sensing conditions to assess the generalizability of the proposed framework more comprehensively. In addition, further investigation will focus on mechanisms better suited to small-object segmentation and on adaptation to other sensing platforms, including satellite imagery and cross-resolution data sources. 

## Figures and Tables

**Table 1:** Comparison of the ISPRS Vaihingen and Potsdam datasets.

Characteristic	Vaihingen	Potsdam
Location	Vaihingen, Germany	Potsdam, Germany
Tile size (pixels)	2494 × 2064	6000 × 6000
GSD (cm/pixel)	9	5
DSM available	Yes	Yes
Number of classes	6	6

**Table 2:** Comparison of different methods on the ISPRS Vaihingen dataset. The best results are shown in bold.

Method	Modality	OA (%)	mIoU (%)	F1 (%)				
				ImSurf	Building	LowVeg	Tree	Car
UNet	Unimodal	89.51	75.13	93.20	93.03	82.04	87.30	74.35
FuseNet	Multimodal	91.04	81.21	94.24	94.50	83.57	89.53	85.40
ACNet	Multimodal	91.71	82.86	94.88	95.39	84.43	89.83	87.79
ESANet	Multimodal	89.70	76.94	93.17	93.18	82.21	88.17	76.24
CMFNet	Multimodal	90.64	80.86	93.97	94.26	82.90	89.01	86.06
ASMFNet	Multimodal	88.68	72.96	92.29	91.66	81.45	87.77	64.51
FTransUNet	Multimodal	89.65	76.47	93.25	93.10	82.45	87.97	74.41
PACSCNet	Multimodal	91.65	83.38	94.85	95.38	84.11	89.79	**89.80**
**Ours**	Multimodal	**92.41**	**84.06**	**95.13**	**95.81**	**85.37**	**89.97**	89.34

**Table 3:** Comparison of different methods on the ISPRS Potsdam dataset. The best results are shown in bold.

Method	Modality	OA (%)	mIoU (%)	F1 (%)				
				ImSurf	Building	LowVeg	Tree	Car
UNet	Unimodal	84.56	76.19	88.84	89.51	82.19	81.37	91.74
FuseNet	Multimodal	88.31	82.00	90.78	93.01	84.99	86.41	94.73
ACNet	Multimodal	89.90	84.61	92.57	95.55	85.87	87.77	95.80
ESANet	Multimodal	85.81	78.01	89.51	90.20	83.63	82.77	91.55
CMFNet	Multimodal	84.80	76.87	89.01	89.09	82.39	81.67	91.75
ASMFNet	Multimodal	85.71	77.42	89.73	90.84	82.77	80.97	91.23
FTransUNet	Multimodal	84.44	75.90	88.44	89.48	81.20	78.88	92.35
PACSCNet	Multimodal	89.99	84.61	92.65	94.51	86.97	87.94	95.69
**Ours**	Multimodal	**90.43**	**85.31**	**92.97**	**95.58**	**87.03**	**88.13**	**96.37**

**Table 4:** Ablation study on the Vaihingen dataset.

Variant	Boundary Prior	Bidirectional	OA (%)	mIoU (%)
(a) Full model (ours)	✓	✓	92.41	84.06
(b) w/o boundary prior	✕	✓	91.42	83.05
(c) w/o bidirectional fusion	✓	✕	91.70	83.35

**Table 5:** Ablation study on the Potsdam dataset.

Variant	Boundary Prior	Bidirectional	OA (%)	mIoU (%)
(a) Full model (ours)	✓	✓	90.43	85.31
(b) w/o boundary prior	✕	✓	89.30	84.18
(c) w/o bidirectional fusion	✓	✕	89.58	84.50

## Data Availability

The aerial imagery and digital surface models (DSMs) used in this study are publicly available through the ISPRS Urban Modelling and Semantic Labelling Benchmark. The datasets for the Vaihingen and Potsdam test sites can be accessed via the official ISPRS 2D Semantic Labeling Contest website at https://www.isprs.org/resources/datasets/benchmarks/UrbanSemLab/semantic-labeling.aspx accessed on 12 May 2026.
